# Integrative methylome and transcriptome analyses identify *LHX3* as an epigenetically downregulated marker with tumor suppressive properties in breast cancer

**DOI:** 10.1371/journal.pone.0357378

**Published:** 2026-10-07

**Authors:** Kokab Farooq, Sheereen Gull, Zubia Farooq, Muhammad Talal Amin, Rana Salman Anjum, Muhammad Hassan Sarfraz, Ajaz Ahmad, Naveed Shahzad

**Affiliations:** 1 School of Biological Sciences, University of the Punjab, Lahore, Pakistan; 2 Shaukat Khanum Memorial Cancer Hospital & Research Centre, Lahore, Pakistan; 3 Faculty of Bioscience Engineering, Ghent University, Ghent, Belgium; 4 Forman Christian College, (A Chartered University), Lahore, Pakistan; 5 Department of Oral Oncology, Roswell Park Comprehensive Cancer Center, Buffalo, New York, United States of America; 6 Nuffield Department of Orthopaedics, Rheumatology and Musculoskeletal Sciences, Botnar Institute of Musculoskeletal Sciences, University of Oxford, Oxford, United Kingdom; 7 Department of Clinical Pharmacy, College of Pharmacy, King Saud University, Riyadh, Saudi Arabia; University of Bonn, Institute of Experimental Hematology and Transfusion Medicine, GERMANY

## Abstract

Breast cancer (BC) remains a major global health concern, with rising incidence and mortality rates. Early diagnosis of BC plays an important role in its prevention. However, number of clinically validated biomarkers that enable accurate and minimally invasive diagnosis are still insufficient. Epigenetic modifications, particularly hypermethylation-mediated inactivation of numerous tumor suppressor genes occur early in tumorigenesis and are detectable even before the clinical onset, making them promising candidates for early diagnosis. Therefore, this study aimed to identify breast cancer-associated epigenetically regulated genes with potential diagnostic and biological relevance. A set of forty genes that displayed both hypermethylation and downregulation in BC were initially identified through bioinformatics analysis of publicly available datasets. Based on Gene Ontology (GO) and Reactome pathway enrichment analyses, eight genes were subsequently shortlisted for experimental analysis. Among these eight genes, *LHX3* was significantly downregulated in both MCF-7 and MDA-MB-231 cells and in all tested BC tissue samples. *LHX3* promoter hypermethylation was found in 80% of tumor tissues, compared to 32% in matched normal tissues. Aberrant *LHX3* promoter methylation was significantly associated with metastasis status (P = 0.0002), with higher methylation in non-metastatic tumors, tumor size ≤20 mm (P = 0.002), and advanced tumor stage (P = 0.005). Given the complex pattern of these associations, findings were interpreted cautiously. Treatment with 5-aza-2’-deoxycytidine reversed promoter hypermethylation and restored *LHX3* expression in MDA-MB-231 and MCF-7 cells. Additionally, ectopic expression of *LHX3* not only reduced migration and proliferation but also induced cell death in BC cells. Our study provided the first evidence of epigenetically induced silencing of *LHX3* and its anti-tumor role in BC. Furthermore, our findings support *LHX3* as a candidate epigenetically regulated biomarker with tumor-suppressive properties in BC; however, independent cohort validation, quantitative methylation assays, and stage/subtype-stratified analyses are required before its diagnostic or prognostic utility can be established.

## Introduction

Breast cancer (BC) is one of the most prevalent cancers globally and stands as the second leading cause of cancer-related deaths in women [1]. Late diagnosis significantly contributes to the high mortality rate of BC, particularly in resource-limited countries like Pakistan. The prevalence of advanced stages is often attributed to insufficient and costly healthcare systems, highlighting the need for more dependable approaches to early-stage tumor diagnosis and therapy monitoring. Conventional diagnostic methods such as mammography, MRI, biopsy, and histological examinations are generally less sensitive, more invasive, and expensive. In contrast, identifying molecular abnormalities in BC presents several advantages. For example, diagnosis based on molecular biomarkers can be implemented at early stages, requires less demanding handling, is cost-effective, facilitates sample processing, and enables prompt delivery of results. Furthermore, for point-of-care (POC) applications, they can be incorporated relatively easily into portable and user-friendly devices [2].

BC development and progression involve specific genetic and epigenetic changes that disrupt the functionality of cancer associated genes [3]. These changes can act as a foundation for developing novel and unique diagnostic and therapeutic methodologies. The increased methylation of promoters is one of the most frequently encountered epigenetic alterations associated with the transcriptional inactivation of various tumor suppressor genes (TSGs) in different types of cancers [3]. Promoter hypermethylation of TSGs occurs at very early stages of carcinogenesis and can be maintained over long periods [4]. However, this change is considered reversible, which makes it a potentially promising target for treatment [5]. The methylation-based transcriptional inactivation of numerous TSGs including *ATM*, *BRCA1*, *BRCA2*, *TP53*, *MLH1*, *CHEK2* [6]. *MGMT, HOXD11, LAMA3, LAMB3, ROBO1 CDH13, CST6, SYK*, *CDKN1C, FOXA2, PGR, RASSF1A, RUNX3*, *TWIST, DAPK, HOXA5, BCL2, DCC, SHISA3* and *HLF* have been documented by recent research [4,7–9]. However, reliable molecular biomarkers that can improve BC detection, classification, or clinical characterization remain limited. Hence, the identification of new DNA methylation biomarkers related to BC would hold significant importance in the advancement of BC diagnostics and therapeutics.

LIM homeodomain genes (LHXs) belong to a subfamily of homeobox genes. They encode proteins featuring two LIM domains (for protein interactions) and one homeodomain (HD) (for binding to the DNA of target genes). These proteins play crucial roles in various biological processes, with their regulation of developmental pathways particularly noteworthy [10]. Recent studies have highlighted the significant involvement of LHXs in cancer development. Among the 12 human LHXs, 10 LIM-HD proteins have been recognized to be linked with diverse cancer types [11]. Additionally, certain LHXs have been proposed as cancer biomarkers for initial diagnostic and prognostic assessment. LHXs serve as either tumor suppressors or oncogenes, based on the specific tumor type. For instance, *LHX2* plays tumor suppressing role in leukemia [12], but in breast and cervical cancer it functions as an oncogene [13,14]. Similarly, the role of *LHX3*, another member of the LHXs family, remains unknown across different cancer types, despite its vital function in pituitary development during early fetal life [15–17]. *LHX3* has been reported as a metastatic oncogene in lung adenocarcinoma [18], liver carcinoma [19,20], and squamous cell carcinoma of head and neck [21]. However, very few studies have explored the role of *LHX3* in BC. Dietrich et al. (2009) documented increased *LHX3* methylation in invasive ductal breast carcinoma (IDC) and ductal carcinoma in situ (DCIS) of preserved breast tissue samples [22]. Conversely, no noticeable difference in *LHX3* expression was observed between BC and normal samples [23]. Nevertheless, these studies have certain limitations, underscoring the need for in-depth analysis of *LHX3* expression, epigenetic regulation, and functional roles to elucidate the diagnostic significance of *LHX3* in BC carcinogenesis.

In the present study, we curated publicly available datasets for genome-wide analysis of differentially expressed and methylated genes in BC. Our analysis identified 40 putative tumor suppressor genes. Among these, *LHX3* showed methylation-based transcriptional inactivation due to promoter hypermethylation in the BC cell line and primary tissues. Moreover, ectopic expression induced tumor suppressor properties in BC cells. For the first time, the tumor suppressor function of *LHX3* in BC is described in this paper. These findings may provide a basis for further validation of *LHX3* as an epigenetically regulated tumor-suppressive candidate with potential diagnostic or therapeutic relevance in BC.

## Methods

### Data acquisition and selection of gene pool

For the comprehensive screening of genome-wide methylation and transcriptome profiles, we utilized the publicly available Gene Expression Omnibus (GEO) repository, known for archiving diverse high-throughput screening data [24]. To curate datasets relevant to our study, we employed strategic queries such as ‘Breast Cancer’ and ‘tumor suppressor,’ and further refined our dataset selection by taking metadata factors such as experimental context, technology employed, and sample details into consideration. We filtered eight microarray datasets for methylation and four for expression (Table 1).

**Table 1:** Expression and Methylation datasets obtained from GEO archives and investigated in this study.

Datasets	Platform		Breast Normal Samples	Breast Tumor Samples	Sample Type	Reference
	Methylation Array	Expression Array				
GSE26349	GPL8490	–	2	17	*CIMP + & CIMP-	[25]
	–	GPL571	11	10		
GSE26990	GPL8490		1	47	Breast tissues	[26,27]
			1	30	Cell lines	
	–	GPL6947	6	6	Cell lines	
GSE20711	–	GPL570	2	88	Breast tissues	[28]
GSE22250	–	GPL570	8	7	Cell lines	[28]
GSE22249	GPL8490	–	8	117	Breast tissues	[28]
GSE33065	GPL8490	–	4	43	Breast tissues	[29]
GSE32393	GPL8490	–	23	114	Breast tissues	[30]
GSE20712	GPL8490	–	2	88	Breast tissues	[28]
GSE31979	GPL8490	–	21	103	Breast tissue	[31]
GSE20868	GPL8490	–	3	3	HCT116 & DKO HCT116 cell lines	[28]

*CIMP: CpG Island Methylator Phenotype.

Raw data from these microarrays was analyzed to curate differentially expressed genes (DEGs) and differentially methylated genes (DMGs) by using web-based GEO2R tool. It is an interactive online tool designed to compare different groups of samples in GEO series and facilitates the identification of differentially expressed genes based on experimental conditions. All non-cancerous samples, including normal breast tissues, normal breast cell lines, and the HCT116 (used as normal methylation standard), were grouped as ‘Normal’. These were compared with cancerous samples, including breast tumor tissues, cancer cell lines, and DKO HCT116 cells (used as standard for hypomethylation), which were grouped as ‘Tumor’.

DEGs were obtained utilizing the Limma R package, employing a criterion of |FC| > 2 for log2FoldChange and a false discovery rate (FDR) cut-off of *P* < 0.05 according to the Benjamini & Hochberg method. For DMGs, statistical significance was determined based on |t| > 2 and a significance level of *P* < 0.05. Eventually, VLOOKUP excel function was used to superimpose DMGs and DEGs to pool out a list of hypermethylated-low expression genes.

### Pathway enrichment and functional analysis

Gene ontology (GO) analysis was carried out on selected gene pool (hypermethylated-low expression genes) using Database for Annotation, Visualization, and Integrated Discovery (DAVID). DAVID is a functional annotation tool, widely used for functional and pathway enrichment analysis to annotate large sets of genes to provide overview of their biological role [32]. Metabolic pathway analysis was performed by Reactome database [33]. Parameters such as minimum genes count of ≥2 and Modified Fisher Exact P-value for gene-enrichment analysis (EASE) score of 0.1 were set by default.

### Patient samples and cell lines

A total of 150 primary breast cancer and corresponding normal mammary tissue samples were collected from Sir Ganga Ram hospital, Lahore, Pakistan. These included 50 formalin-fixed paraffin-embedded (FFPE) samples and 100 fresh surgical specimens prior to any therapeutic intervention. Samples were either snap-frozen in liquid nitrogen or preserved in RNA later™ (Invitrogen) until nucleic acid extraction. Tissue processing and section cutting for H&E staining were carried out for histopathological assessment. Clinicopathological data such as menopausal status, age at diagnosis, tumor size, histological grade and tumor staging were registered.

Two BC cell lines: MCF-7 and MDA-MB-231 were acquired from the repository of the School of Biological Sciences (SBS), University of the Punjab Lahore, Pakistan. These cell lines were cultured and maintained according to the guidelines for animal cell culturing provided by ATCC. To ensure uniform culturing conditions during downstream bioassays, both cell lines were maintained in Dulbecco’s Modified Eagle Medium (DMEM) instead of ATCC recommended EMEM media for MCF-7 cells and Leibovitz’s L-15 medium for MDA-MB-231 cells. DMEM was supplemented with 1% penicillin-streptomycin (Gibco) and 10% Fetal Bovine Serum (FBS) (Gibco). Cells were incubated at 37 °C in the presence of 5% CO_2_ and humidity.

### Expression analysis by qRT-PCR

The total RNA was extracted from tissues and cell lines using Trizol (Invitrogen) method [34]. The extracted RNA was treated with DNase to remove potential genomic DNA contamination. The concentration of RNA was determined by using NanoDrop 2000 spectrophotometer (Thermo Fisher Scientific) and A260/280 ratio ranging from 1.8 to 2.0 and A260/230 ratio ranging from 2.0 to 2.2 were considered acceptable for its purity. Subsequently, 1 µg of RNA from each sample was transformed into cDNA utilizing the RevertAid first strand cDNA Synthesis kit (Thermo Fisher Scientific) according to the kit’s instructions. A no-reverse-transcriptase (–RT) control was also included to assess genomic DNA contamination. The mRNA expression of shortlisted genes was analyzed using SYBR Green assay in PicoReal SW 2.2 quantitative real-time PCR system (Thermo Fisher Scientific). The cycling protocol utilized was as follows: 95 °C for10min, [95 °C for 30sec, 58 °C for 30sec, 72 °C for 30sec] 35X and final extension at 72 °C for 5 min. A list of genes and their primer details are provided in Table 2. Quantification of the results was performed by using the 2^−ΔΔCt^ method 2 − ΔΔCt method [35]. Relative change in mRNA level between breast tumor and normal samples was presented as fold change after normalization with reference gene β-actin. Genes were labeled as “downregulated” in tumor tissues when their expression level was less than 1/3^rd^ of the corresponding normal tissues.

**Table 2:** List of genes and their corresponding primers used in this study.

Primers	Forward primer (5’-3’) (F)	Reverse primer (5’-3’) (R)	Amplicon size (bp)	NCBI Gene Accession Number	Position	Exon F, R
**qPCR Primers**						
*B-actin*	GATGAGATTGGCATGGCTTT	CACCTTCACCGTTCCAGTTT	100 bp	NM_001101.5	chr7:5566779–5570232	4, 4
*LHX3*	GTGCAGGTTTGGTTCCAGAA	CTCCTGAACGCTGTCCTTGT	141 bp	NM_014564	chr9:139088096–139095035	4, 5−5
*KLK10*	ACTGCGGAAACAAGCCACTG	GGGATGGACAACAGAGCGAG	101 bp	NM_002776.4	chr19:51012744–51020026	2-3, 3
*BHMT2*	CTGGGGGTCAGGTACATTGG	ATACTCCCTTCGAGCCCTTG	183 bp	NM_017614.4	chr5:79069724–79090074	6, 6-7
*HLF*	ATCAGACCAGGTCAGCTGTT	TGGCTTCAGTTCTTCCTCAG	180 bp	NM_002126	chr17:55264960–55325187	2-3, 3
*CDC14B*	ATTCCTTTCAGAGATGCTGCC	TTAAGGAAGCCATACTGCATTG	101 bp	NM_033331	chr9:96500113–96619843	4, 5-6
*AKAP12*	GTCAGCTGTCCACCATCAAT	CCATCATCTGTGATGTCGTG	235 bp	NM_005100	chr6:151239967–151358559	2, 3
*CCND2*	GGAGGTCTGTGAGGAACAGA	GGAGTTGTCGGTGTAAATGC	199 bp	NM_001759	chr12:4273762–4305352	1-2, 2
*MAP3K7*	TCCAGTGTGTCTTGTGATGG	ATAAGCCACTCCTTGGGAAC	136 bp	NM_145332	chr6:90513580–90587072	3-4, 5
**MSP primers**						
*LHX3* methylation	TCGTTTTCGGGTCGGGTAC	GAAACCGAAAATCGCGAAAACC	108 bp	NM_014564	Chr9:136,203,346−136,203,238 bp	
*LHX3* Unmethylation	TTTGTTTTTGGGTTGGGTATGTG	GAAACCAAAAATCACAAAAACCAAC				

### Methylation analysis by MSP

After DNA extraction using the method explained by Naz et al. (2021) [36], 2 µg of total DNA from each sample was modified by giving sodium bisulfite treatment following the protocol provided by EZ DNA Methylation-Gold™ Kit (Zymo Research). Promoter sequence of candidate gene (*LHX3)* was retrieved from Eukaryotic Promoter Database (EPD) [37] and submitted to MethPrimer to predict CpG islands (S1 Fig) and to design methylation-specific PCR (MSP) primers. Two *LHX3* primer sets were designed to selectively amplify CpG sites in bisulfite-converted DNA based on their methylation status. The “methylation primer set” (M) exclusively amplified methylated CpG sites, whereas the “unmethylation primer set” (U) specifically targeted unmethylated CpG sites (Table 2). The specificity and quality of the primers were validated by commercially available human methylated and unmethylated DNA controls (Zymo Research™). Each MSP reaction was comprised of 1X reaction buffer, 25mM MgCl_2_, 2.5mM dNTPs, 1μM of each primer, 200ng of bisulfite converted DNA template, 5U of Hot start Taq polymerase (Thermo Fisher Scientific), and water for volume makeup up to 25μl.

Following touchdown PCR profile was employed: 95 °C for 4 min, [94 °C for 1 min, 67 °C for 1 min with 1 °C decrease in each cycle, 72 °C for 1 min] 11X, [94 °C for 30sec, 57 °C for 30sec, 72 °C for 30sec] 35X and final extension at 72 °C for 5 min. The PCR products were observed under ultraviolet (UV) light following electrophoresis on a 2% agarose gel. The presence of a DNA band in the methylated or unmethylated reaction indicated the presence or absence of promoter methylation at the target site.

### *In-silico* expression and methylation analysis of *LHX3*

The difference in *LHX3* expression and methylation between the normal breast tissues and various subtypes of BC were also investigated by *in-silico* analysis. Data collection and analysis was performed following the procedure described by Shahzad et al. (2020) [9]. In brief, FPKM values of *LHX3* expression quantification by RNA-Seq were obtained from TCGA-BRCA for invasive basal carcinoma (IBC), invasive lobular carcinoma (ILC), and invasive ductal carcinoma (IDC), with a fold change threshold of <1. However, for normal breast tissues (NBr), FPKM values for *LHX3* were missing or undetected, necessitating the acquisition of RNA-seq data from the Genotype-Tissue Expression (GTEx) portal instead of Genomic Data Commons (GDC). To compare methylation differences, beta values (with significance level of *P* < 0.01) for *LHX3* were collected from GDC, as previously described.

### De-methylation treatment for expression and methylation analysis

Methylation-based transcriptional inactivation of *LHX3* was further confirmed through *in-vitro* demethylation treatment. BC cell lines were plated in 6-well plates at a concentration of 1X10^6 cells/ml for 24h. Subsequently, cells were exposed with methyl inhibitor 5-Aza-2′-deoxycytidine (Aza, Abcam) at 10μM and 20μM concentrations for 72h. Cultural media was replaced daily with freshly prepared drug-containing media. Demethylation was confirmed by MSP, and the effect of reduced methylation on restoring *LHX3* expression was determined using qRT-PCR.

### Ectopic expression of *LHX3* in BC cells and effect on malignant behavior

An ORF clone of *LHX3* in mammalian expression vector (pcDNA3.1-LHX3) was obtained from GenScript Biotech, Singapore and confirmed by restriction digestion with HindIII and BamHI enzymes. MDA-MB-231 and MCF-7 cells were plated at a concentration of 1X10^4 cells/cm^2^ in 6-well plate to attain 50% confluency after 24h. cells were then transiently transfected with 2 μg of either an empty vector pcDNA3.1 as a transfection control or pcDNA3.1-LHX3 vector with the help of transfection reagent Lipofectamine 3000 (Invitrogen). Non-transfected cells were included as a negative control in each assay. Transfection efficiencies and *LHX3* overexpression was measured by qRT PCR by taking cells pellet at various time points (0, 24, 48, and 72 hours) following transfection.

A series of bioassays (such as proliferation, colony formation, apoptosis, migration, and invasion assays) were conducted by following the protocols described by Farooq et al (2025) [7] to investigate the impact of *LHX3* ectopic expression on the malignant characteristics of BC cells.

### Statistical analysis

All experiments were conducted in duplicates across three independent settings. Significance was defined as *P* < 0.05, and findings were presented as mean±standard deviation or percentage. For statistical analysis and graphical representation of the data, GraphPad Prism version 8 was used. Differences between the groups (normal and tumor; untransfected and transfected) were assessed using the student’s t-test. The Chi-square model assessed the significant correlation between *LHX3* methylation and clinicopathological characteristics among 150 BC patients.

### Ethics approval and consent to participate

All breast cancer tissue samples and associated clinicopathological data were retrospectively collected with written informed consent from the participants. The samples and clinical data were accessed for research purposes between 23/10/2017 and 21/12/2018, following approval by the Institutional Bioethics Committee, University of the Punjab, Lahore (Bioethics/094; 13 October 2017). All experiments and data collection were conducted in accordance with institutional ethical guidelines.

## Results

### Gene pool screening and shortlisting

Using methylation microarray data from eight different datasets, we identified a substantial cohort of differently methylated genes (DMGs). Among these DMGs, 1690 displayed hypermethylation, with at least a two-fold increase in methylation in tumor samples as compared to normal samples. Additionally, from the analysis of differentially expressed genes (DEGs) in gene expression microarrays, we found 718 downregulated genes. Through an overlap analysis between the hypermethylated gene set (1690 genes) and the downregulated gene set (718 genes), we identified 40 genes as hypermethylated-downregulated genes (Fig 1a) (S1 Table).

**Fig 1:**
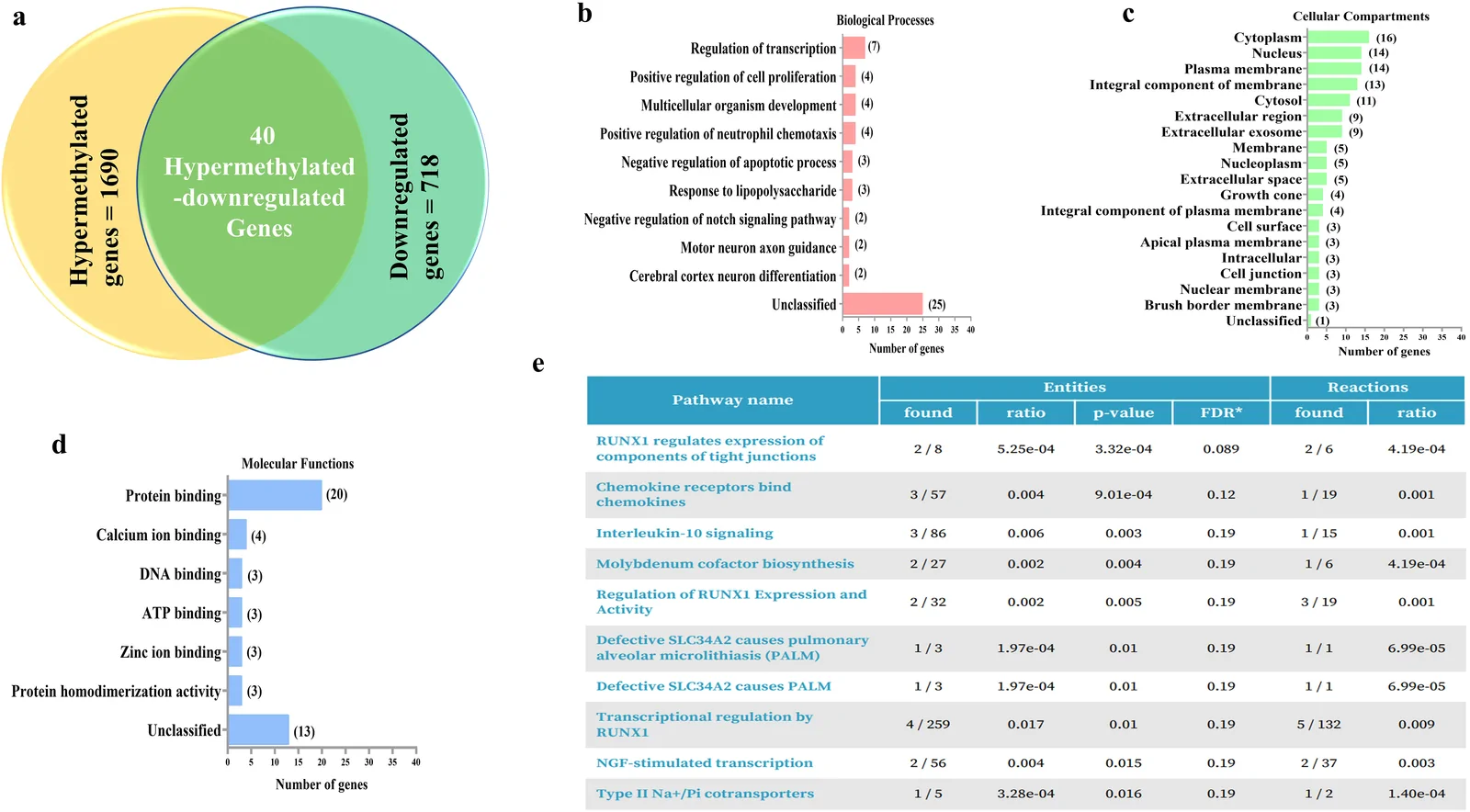
Screening and shortlisting of genes. (a) A List of 40 hypermethylated-downregulated genes was obtained by superimposing the downregulated and hypermethylated gene sets screened from the GEO database. Enrichment analysis for the identified genes was performed to annotate their biological functions. Bar graphs represent categories of GO terms such as (b) Biological processes, (c) Cellular compartments, and (d) Molecular functions. The x-axis indicates the frequency of the genes in the corresponding categories. The Y-axis represents the categories of gene functions. The number of genes/hits is mentioned against each bar. (e) Pathway analysis by Reactome displays the top 10 pathways regulated by the hypermethylated-downregulated gene set. *FDR = False discovery rate.

Functional annotation through Gene Ontology (GO) analysis showed that 40 hypermethylated-downregulated genes were involved in various biological processes. The top category included seven genes associated with transcriptional regulation and processes related to cellular transformation (Fig 1b). Cellular localization analysis showed that the majority of these genes (n = 16) were localized in the cytoplasm (Fig 1c), while 20 genes were implicated in protein binding (Fig 1d). Fold enrichment values, gene symbols, and *P* values are provided in S2 Table. Within the Reactome database, 30 out of the 40 genes were identified, with the majority being involved in pathways related to gene expression. We selected the top 10 most relevant pathways based on significance threshold of *P* < 0.05 (Fig 1e).

As a result of these findings, we shortlisted eight genes (*BHMT2, HLF, LHX3, KLK10, CDC14B, AKAP12, CCND2,* and *MAP3K7*) for further experimental analysis. Detailed information about these genes can be found in S3 Table. These genes were selected based on their hypermethylation and downregulation characteristics and undergone further validation.

### *LHX3* is downregulated in BC cell lines and tissues

Initially, qRT-PCR was performed to assess the expression levels of the eight selected genes in both BC cells and tissues, in comparison to normal tissue samples. Among these genes, *CDC14B* exhibited consistent upregulation in all BC cells and tissues. Conversely, *MAP3K7* showed decreased expression levels in MCF-7 cells, while displaying elevated expression in MDA-MB-231 cells and tumor tissues. *AKAP12* expression levels did not significantly differ between normal and cancer cells. However, five genes *LHX3, HLF, BHMT2, KLK10*, and *CCND2* displayed more than a two-fold downregulation in BC cells and tissues compared to the corresponding normal tissues (Fig 2a & 2b).

**Fig 2:**
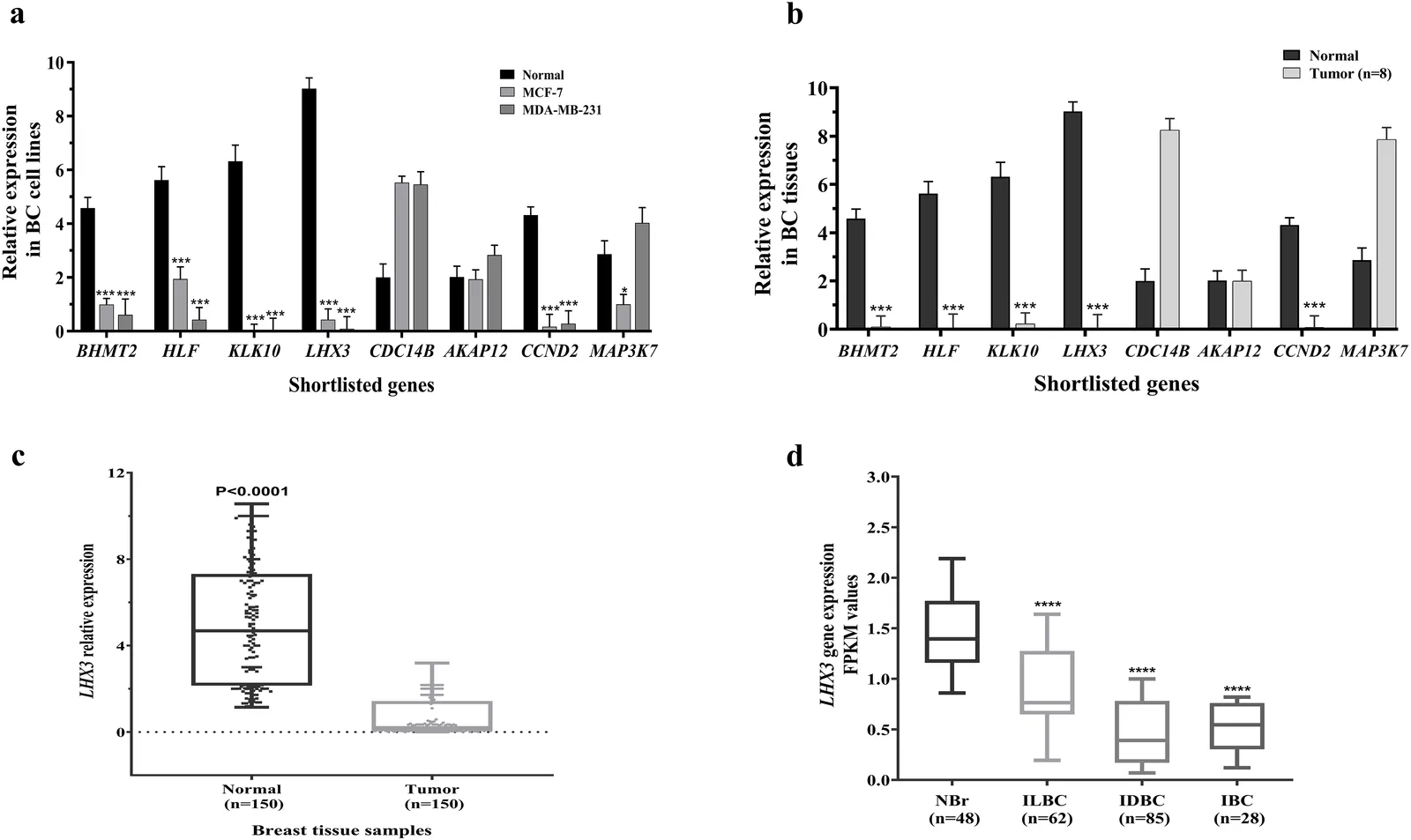
Expression analysis by qRT-PCR. (a) Relative expression analysis of shortlisted genes in BC cell lines and (b) BC tissues (n = 8) compared with the mean expression of 8 normal breast tissues. Multiple t-tests indicate statistical significance. (c): Box-plot diagram of LHX3 gene expression in breast tumor (n = 150) compared with adjacent normal tissue (n = 150) samples. A two-tailed paired t-test indicates statistical significance. (d) In-silico analysis of LHX3 gene expression in normal and various subtypes of BC. NBr: Normal Breast Tissues, ILBC: Invasive Lobular Carcinoma, IDBC: Invasive Ductal Carcinoma, and IBC: Invasive Basal Carcinoma. Two-tailed Mann-Whitney test P-values indicate statistical significance. P < 0.033, **P < 0.002, ***P < 0.001, ****P < 0.0001). Graphs show the mean of three technical repeats. S.D = Error bars.

Remarkably, *LHX3* exhibited a particularly profound downregulation of at least 8-fold compared to other genes. Consequently, *LHX3* became our primary target for biomarker validation experiments. Subsequent investigations involved a large cohort of breast tissues (n = 150 paired samples) to establish statistical significance. As expected, the analysis of *LHX3* gene expression in this cohort demonstrated significant downregulation (*P* = 0.0001) in breast tumor samples (Mean 0.63 ± 0.8) compared to normal tissues (Mean 5 ± 2.8) (Fig 2c). Similarly, *in-silico* gene expression analysis of BC tissues from TCGA-BRCA revealed a statistically significant decrease in *LHX3* mRNA levels across various tumor subtypes (*P* < 0.0001). The most significant downregulation was observed in invasive ductal carcinoma (IDBC: Mean 0.45 ± 0.3), followed by invasive basal carcinoma (IBC: Mean 0.53 ± 0.2), and invasive lobular carcinoma (ILBC: Mean 0.87 ± 0.4) when compared to expression values from GTEx for normal breast tissues (NBr: Mean 1.46 ± 0.3) (Fig. 2d).

### *LHX3* promoter is hypermethylated in BC cells and tissues

Our next step involved a qualitative methylation analysis of the *LHX3* promoter using Methylation-Specific Polymerase Chain Reaction (MSP). We verified the specificity, sensitivity, and efficiency of the MSP primers (“M” and “U”) through methylated and unmethylated controls, confirmed by the presence of distinct, bright DNA bands. Both MCF-7 and MDA-MB-231 cell lines displayed methylated CpGs, consistent with the DNA band pattern observed in the methylated control (Fig 3a).

**Fig 3:**
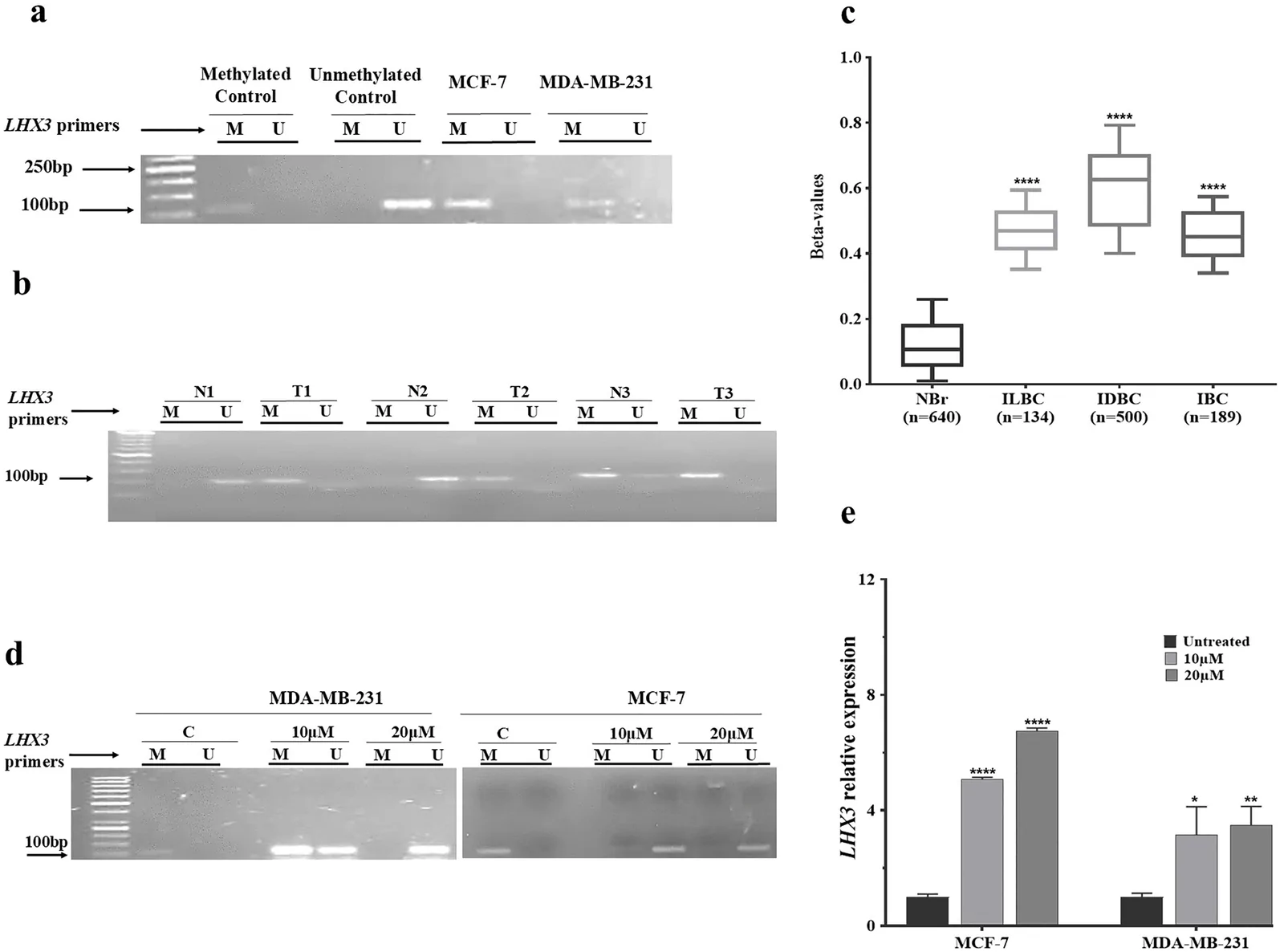
Methylation analysis by MSP. (a) MSP for LHX3 in BC cell lines in comparison with methylated and unmethylated controls. (b) Representative MSP of LHX3 in Breast tumors (T) and adjacent normal tissues (N) samples. (c) Box-plot diagram of LHX3 methylation values obtained from TCGA BRCA for paired breast tissue samples. Paired t-test indicates significantly increased methylation in tumor samples as compared to normal tissues. (d) In-silico analysis of LHX3 methylation in breast normal and subtypes of BC. Two-tailed Mann-Whitney test P-values indicate statistical significance (P = 0.0001). (e) Effect of demethylation by various conc. of Aza on LHX3 gene expression observed in BC cell lines. (f) Demethylation led to the restoration of the expression of LHX3 in both cell lines. An unpaired, two-tailed t-test indicates statistical significance. Graphs show the Mean of three technical repeats. S.D = Error bars.

In BC tissues, we detected methylation at the target CpG site in 80% of the samples (120 out of 150). Conversely, in 30 tumor samples, the “U” primers amplified, indicating the absence of methylation in the target region. Moreover, in 102 out of 150 normal breast tissues, we observed no bands when amplified with the “M” primer, but sharp bands appeared when “U” primers were used. This suggests the absence of promoter methylation in 68% of normal tissues, while 32% of normal samples (48 out of 150) exhibited partial methylation at the *LHX3* target region. The difference in methylation frequency between BC and normal tissue was statistically significant (*P* = 0.00001) (Fig 3b). *In-silico* analysis methylation arrays also showed increased methylation in various BC types. Beta values for *LHX3* obtained from analysis demonstrated increased methylation in invasive ductal breast cancer (IDBC) (Mean 0.6 ± 0.12), invasive lobular breast cancer (ILBC) (Mean 0.47 ± 0.07), and invasive basal carcinoma (IBC) (Mean 0.45 ± 0.07) when compared to normal breast tissue (NBr), where methylation values were significantly lower (Mean 0.12 ± 0.07) (Fig 3c).

We additionally explored the association between *LHX3* methylation and clinicopathological features in 150 BC patients. Aberrant promoter methylation of *LHX3* was significantly associated with metastasis status (P = 0.0002), tumor size (P = 0.002), and TNM stage (P = 0.005). *LHX3* methylation was more frequent in tumors without distant metastasis than in metastatic tumors, while also being enriched in advanced TNM-stage tumors. While no significant associations were observed with other parameters, *LHX3* methylation was observed in 83% of patients with positive nodal involvement, 86% of patients below 50 years of age, and 83% of post-menopausal patients. Ductal carcinoma patients accounted for 83% of those with *LHX3* methylation (Table 3).

**Table 3:** Association between *LHX3* promoter methylation and clinicopathological features of the BC samples.

Characteristics	Cases n = 150	*LHX3* Promoter Methylation		^*^P value
		Present n = 120	Absent n = 30	
**Age at diagnosis**				
≥ 50	78 (52%)	58 (75%)	20 (25%)	(0.07)
<50	72 (48%)	62 (86%)	10 (14%)	
**Menopausal status**				
Pre	29 (19%)	20 (69%)	9 (31%)	(0.09)
Post	121 (81%)	100 (83%)	21 (17%)	
**Tumor size**				
≤20 mm	78 (52%)	70 (90%)	8 (10%)	**(0.002)**
>20 mm	72 (48%)	50 (69%)	22 (31%)	
**Histological type**				
Non-ductal	42 (28%)	30 (71%)	12 (29%)	(0.10)
Ductal	108 (72%)	90 (83%)	18 (17%)	
**Nodal involvement**				
Negative	90 (60%)	70 (78%)	20 (22%)	(0.4)
positive	60 (40%)	50 (83%)	10 (17%)	
**Metastasis**				
Yes	33 (22%)	19 (58%)	14 (42%)	**(0.0002)**
No	117 (78%)	101 (86%)	16 (14%)	
**TNM stage**				
Early I/II	48 (32%)	32 (67%)	16 (33%)	**(0.005)**
Advance III/IV	102 (68%)	88 (86%)	14 (14%)	

Data are reported as frequencies and percentages. Differences between proportions were assessed by *Chi square test. Significant at *P*<0.05.

### Aza treatment reduced methylation and restored *LHX3* expression

To investigate whether the decrease in *LHX3* expression resulted from hypermethylation of its promoter region, MDA-MB231 and MCF-7 cells were exposed to 10 and 20μM of the DNA methyltransferase inhibitor 5-aza-2′-deoxycytidine (Aza, Abcam). This treatment significantly reduced the methylation of the *LHX3* promoter, as confirmed by MSP (Fig 3d). Conversely, a significant increase in *LHX3* expression was observed in both cell lines as compared to the non-treated control. Expression levels showed a gradual, dose-dependent increase, with MCF-7 displaying more pronounced upregulation than MDA-MB-231 (Fig 3e). In summary, our findings suggested that promoter methylation is a primary reason for the silencing of *LHX3* in BC cells.

### Ectopic expression of *LHX3* inhibited tumorigenesis in BC cells

To explore the involvement of *LHX3* in tumor development, we introduced *LHX3* ectopically into MCF-7 and MDA-MB-231 cell lines. Analysis of qRT-PCR revealed a gradual increase in *LHX3* expression levels in both cell lines transfected with the LHX3-expressing vector (pcDNA3.1-LHX3). The highest expression was observed at 72 hours after transfection. Meanwhile, the expression of β-actin remained unchanged in these transfected cells (Fig 4a).

**Fig 4:**
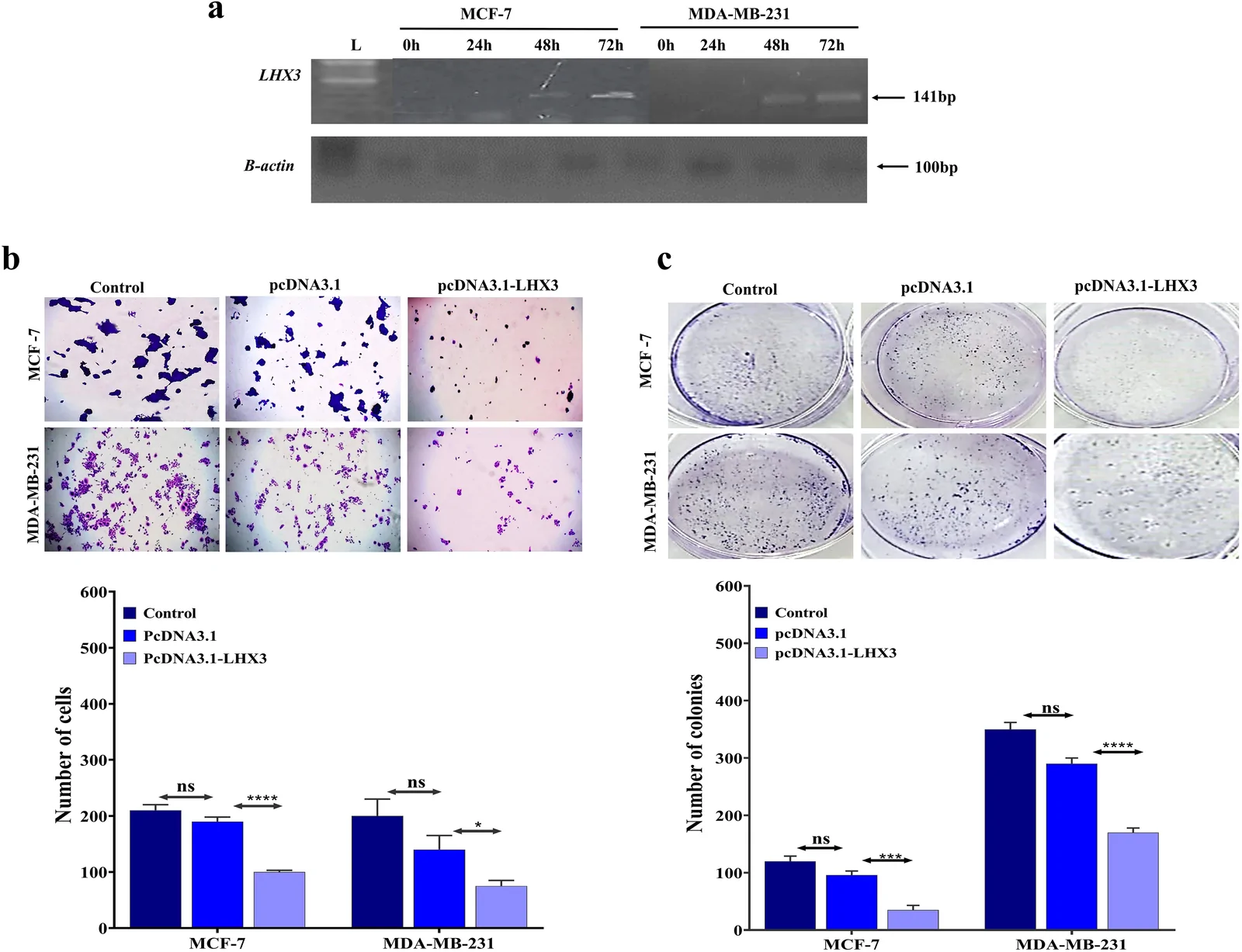
Transfection optimization and bioassays to explore the anti-tumorigenic role of LHX3. (a) Representative gel images of RT-PCR (b) (upper panel) post-transfection images of Crystal violet assay represented a reduction in proliferation in both cell lines. (10X magnification). (lower panel) Graphical representation of no. of cells counted after staining with crystal violet. (c) (upper panel) Induced LHX3 expression significantly reduced the colony formation potential of BC cells. The appearance of discrete and distinguishable colonies was captured after 2 weeks of post-transfection seeding of the cells. (magnification 4X). (lower panel) Graphical representation of no. of colonies counted after staining with crystal violet. Graphs show the Mean of three technical repeats. A two-tailed t-test indicates statistical significance between the cells transfected with empty vector control (pcDNA3.1) and LHX3-expressing vector (pcDNA3.1-LHX3). The difference between non-transfected cells and cells transfected with empty vector control is not significant (ns). S.D = Error bars.

We further assessed the proliferation and clonogenic survival of LHX3-transfected BC cells. The results of the Crystal violet assay revealed that cancer cells proliferation was significantly reduced by the ectopic expression of *LHX3*, with a 52% reduction in MCF-7 and a 54% reduction in MDA-MB-231 cells transfected with pcDNA3.1-LHX3 vector compared to cells transfected with the empty vector (pcDNA3.1) (Fig 4b). Similarly, colonies formed by MCF-7 and MDA-MB-231 cells transfected with the LHX3-expressing vector were notably smaller and fewer (mean 35 ± 8 and mean 170 ± 8, respectively) than those formed by cells transfected with the empty vector (mean 96 ± 7 and mean 290 ± 10, respectively). Ectopic expression of *LHX3* significantly reduced cell survival ability by 64% in MCF-7 and by 42% in metastatic MDA-MB-231 cells (Fig 4c). These results are based on an average of three independent experiments, ensuring the robustness of the findings.

Additionally, we examined whether *LHX3* overexpression impacts the migratory and invasive capabilities of BC cells, ultimately influencing their behavior. The anti-invasive potential of LHX3-transfected cells was observed through a transwell assay. The lowest invasion was observed by MCF-7 (46%), followed by MDA-MB-231 (43%), in comparison to the empty vector control (Fig 5a). The migratory capacity of transfected cells was analyzed by using a wound healing assay. Given the metastatic nature of cancer cells, wound closure in the empty vector-transfected MCF-7 and MDA-MB-231 cells reached 76% and 84%, respectively. In contrast, overexpression of *LHX3* led to only 24% and 20% wound closure in MCF-7 and MDA-MB-231 cells (Fig 5b). Altogether, our data demonstrated that *LHX3* has significant potential to prevent proliferation and metastasis in BC cells.

**Fig 5:**
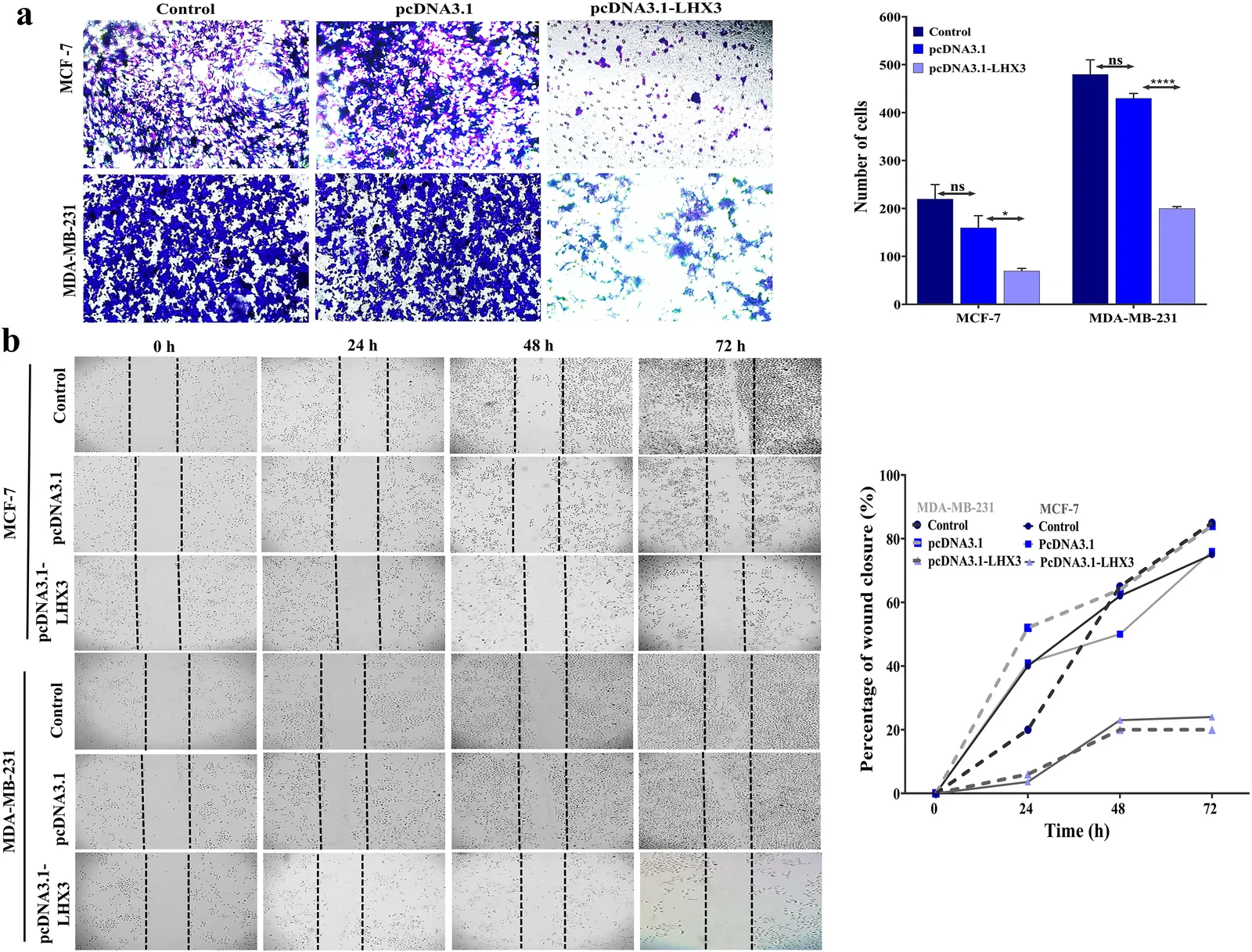
LHX3 overexpression reduced metastatic ability of BC cells (a) (Left) Representative images of Transwell invasion assay in non-transfected control and cells transfected with empty vector and LHX3-vector. (10X magnification). (Right) Graphical representation of the no. of cells that passed through the membrane after transfection. MDA-MB-231 displayed more invasiveness than MCF-7. (b) (Left) Post-transfection images of transfected BC cells, while non-transfected cells serve as a control, illustrating cell motility and wound healing at various time points. (magnification 4X). (Right) Percentage of wound healing in BC cells relative to controls at 0, 24, 48, and 72 hours. Graphs show the Mean of three technical repeats. A two-tailed t-test indicates statistical significance. ns = not significant. S.D = Error bars.

### Ectopic expression of *LHX3* induces apoptosis in BC cells

Finally, we investigated whether *LHX3* promotes apoptosis in BC by inducing DNA damage. Hoechst staining revealed nuclear fragmentation in LHX3-transfected cells, as shown in Fig 6a, where bright blue hyperchromatism indicates DNA damage. Unrepaired DNA damage often leads to programmed cell death, which was confirmed by flow cytometric analysis. *LHX3* overexpression caused cell growth arrest in the G1 phase and decreased the number of live cell populations, as indicated by the G1 peak. Additionally, the number of dead cells increased, as observed by the elevated peak of the sub-G1 population, indicating apoptosis (Fig 6b). In empty vector-transfected cells, the number of apoptotic populations was low, as deduced from the smaller peak of the sub-G1 population.

**Fig 6:**
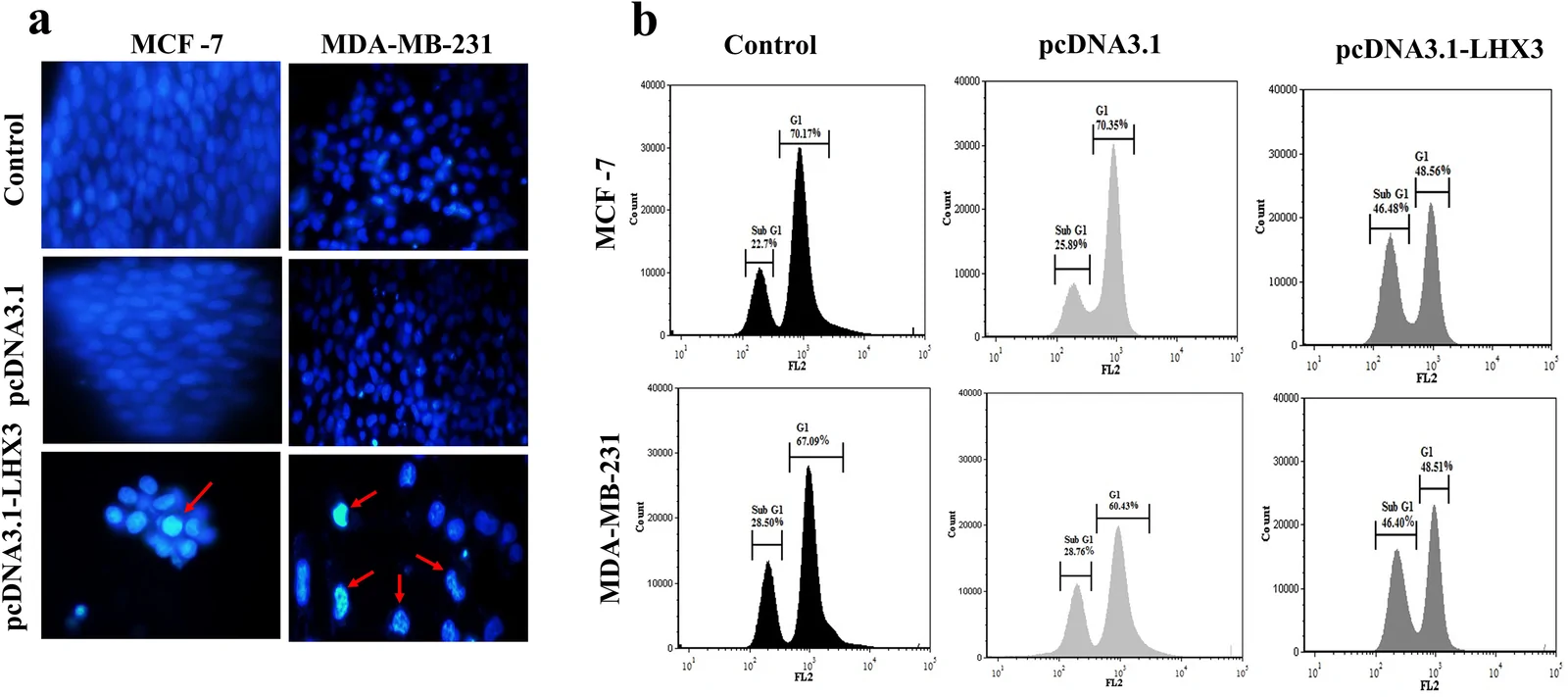
LHX3 overexpression-induced apoptosis in BC cells. (a) Morphological changes visualized by fluorescence microscopy after Hoechst staining. Nuclear thickening and nuclear fragmentation increased significantly with time in LHX3 overexpressing cells, indicated by the red arrow. (magnification 40X). (b) Representative data from one of the three experiments of PI staining followed by flow cytometry. Abundant apoptosis (Sub-G1 peak) of LHX3 and decreased no. of the live cell population (G1 peak) is observable in LHX3 overexpressing cells.

## Discussion

Diagnosis of BC in its early stages remains a significant challenge in the field of oncology. Existing BC diagnostic techniques, including gold standard mammography, encounter specific constraints. These include high costs, time-consumption, limited ability to detect small cancers, and their inappropriateness for use in young women [38]. Consequently, there is an immediate requirement to develop a highly sensitive and swift method for early detection of BC. Researchers have recently concentrated on the identification and development of precise epigenetic biomarkers for early BC detection. These epigenetic biomarkers, specifically tumor-specific DNA methylation alterations, offer several advantages due to their stability, heritability, and reversibility [39]. With the aim of identifying epigenetic biomarkers relevant to BC detection and characterization, we curated online available BC datasets and shortlisted 40 putative tumor suppressor genes showing both hypermethylation and downregulation. These candidates underwent rigorous shortlisting based on their functional annotations and potential involvement in cancer-related pathways, ultimately yielding eight genes of particular interest. Among these, *LHX3* has shown promising potential as a tumor suppressor and has emerged as a candidate epigenetically regulated biomarker in BC.

Integrative genomic analysis has played a crucial role in unraveling key processes associated with cancers. Through this approach, several novel tumor suppressor genes have been identified [40]. Out of the shortlisted 40 putative tumor suppressor genes, the expression of 8 genes was initially verified via quantitative polymerase chain reaction (qPCR). We observed some disparities between our bioinformatics predictions and experimental results for the expression of certain genes. qPCR data indicated an upregulation of *CDC14B* in both cell lines and tissue samples. Similarly, *MAP3K7* exhibited upregulation in all tissue samples and the MDA-MB231 cell line, but not in the MCF-7 cell line. Conversely, *AKAP12* displayed no significant differential expression. The remaining genes, including *LHX3*, aligned with the findings from the bioinformatics analysis. These discrepancies could potentially be attributed to various factors, such as sample size, sample stratification bias, environmental and genetic variations, statistical power, differences in analysis techniques (microarrays vs. qRT-PCR), and data preprocessing and normalization methods.

It has previously been identified that certain genes can play both oncogenic and tumor suppressor functions in different cancers. These “double agents” share functional and intracellular distribution similarities and dissimilarities with both TSGs and Oncogenes making them high-value targets for development of novel cancer therapies [41]. It is worthwhile to note here that about half (41/83) of such genes are known transcription factors [41]. LIM homeobox genes (LHXs) are transcription factors that have been recently demonstrated to have a critical role in neoplastic transformation and cancer progression [11]. The LHXs function as cancer suppressors or promoters, depending on the specific cancer status and types [41]. Additionally, certain LHXs play a dual role in different tumor types. For example, *LHX2* functions as a TSG in T-cell acute lymphoblastic leukemia [12], while it acts as an oncogene in breast and cervical cancer [13,14]. Similarly, *LHX4* exhibited oncogenic properties in colorectal cancer and tumor-suppressing effects in liver cancer [42,43]. *LHX3* was identified to promote the proliferation of cells in lung adenocarcinoma [18], hepatocellular carcinoma [19,20], and head and neck squamous cell carcinoma [21]. Nevertheless, information concerning the function and mechanism of *LHX3* in BC remains limited. Our data demonstrated a significant decrease in the expression of *LHX3* in two BC cell lines (MCF-7 and MDA-MB-231) and most of the BC tissues, as compared to corresponding normal breast tissues. Our *in-silico* analysis further supported these findings by revealing diminished expression of *LHX3* across various subtypes (IBC, IDBC, ILBC). Our results are apparently in disagreement with a recent similar study where authors carried out a detailed analysis of the potential role of LHX family genes in BC utilizing Cancer Genome Atlas (TCGA) data and didn’t observe any significant difference in the expression of *LHX3* between normal and cancerous samples [23]. However, this apparent discrepancy could be because of the difference in sample stratification strategy adopted by the authors of published report, suggesting *LHX3* downregulation could be BC subtype specific. Furthermore, this study also suggested a link between DNA methylation and *LHX3* expression in luminal BC, further supporting our findings. Altogether, these results suggest a potential tumor suppressor role of *LHX3* in BC, which contrasts with the previously described oncogenic function of *LHX3* in other cancer types. To gain further insight, we utilized the TCGA to compare the expression of *LHX3* in different types of cancers, including BC. Our analysis revealed that expression of *LHX3* was notably downregulated in breast tumor tissues in contrast to its elevated expression in head and neck carcinoma, hepatocellular carcinoma, and lung carcinoma (data not shown). This stark difference in *LHX3* expression levels between BC and other carcinomas underscores the intricate and context-dependent nature of *LHX3*’s involvement in tumorigenesis. Earlier studies have identified several LHX genes, such as *LHX4*, *LHX6*, and *LHX9*, that act as tumor suppressors [44–46]. Hence, it is reasonable to speculate that *LHX3* might operate in a comparable manner.

Aberrant DNA methylation patterns, particularly within promoter regions, are recognized as significant drivers of gene expression. Promoter hypermethylation plays a central role in the transcriptional silencing of TSGs and constitutes a critical factor in the initiation and progression of various cancer types, including BC [4]. We observed hypermethylation of the LHX3 promoter in 80% (120/150) of the BC tissues. A recent study also reported hypermethylation of LHX3 in two formalin-fixed breast tissue samples [22]. Notably, 32% of the normal samples showed partial methylation in our study. This methylation in matched adjacent normal tissues may reflect tumor-cell admixture during sampling, field cancerization effects, or baseline methylation, and therefore limits the interpretation of LHX3 methylation as a definitive standalone diagnostic marker without further quantitative validation. Furthermore, in-silico analysis of methylation arrays indicated higher methylation levels in various BC types. The mean *LHX3* methylation levels (Beta values) were calculated as 0.6 ± 0.12, 0.47 ± 0.07, and 0.45 ± 0.07 for IDBC, ILBC, and IBC, respectively, which were significantly higher compared to normal breast tissue (mean 0.12 ± 0.07). These findings suggest that the *LHX3* promoter hypermethylation is a prevalent occurrence in BC, extending beyond a particular subtype.

To establish the relationship between the hypermethylation of *LHX3* promoter and transcriptional inactivation of *LHX3* in BC, we administered 5’-aza-2’-deoxycytidine (Aza), a widely recognized demethylating agent, to BC cell lines. Our results revealed the restoration of *LHX3* expression in these BC cell lines, emphasizing the role of *LHX3* promoter hypermethylation in its suppression. This association was further supported by the fact that the majority of tissues exhibiting downregulated expression showed hypermethylation. The observed association between *LHX3* downregulation and hypermethylation of the promoter CpG target site was statistically significant (*P* = 0.00001) (Table 4). Notably, only 6% of the BC tissues displayed downregulation without a corresponding increase in methylation, suggesting the involvement of additional genetic factors in *LHX3* gene silencing [47]. Similar correlations between gene expression and hypermethylation were also observed in our *in-silico* analysis.

**Table 4:** *LHX3* expression correlation with promoter methylation in BC tissue samples.

Expression				
**Significance level *P*** ^*^ **< 0.05**		Increased n (%)	Unchanged n (%)	Decreased n (%)
**Methylation**	Increased	9 (6%)	18 (12%)	93 (62%)
	Unchanged	15 (10%)	6 (4%)	9 (6%)

*Chi-square test of independence: X2 (2, N = 150) = 35.4, *P*=0.00001.

The tumor suppressor role of *LHX3* was further confirmed by ectopic expression of *LHX3* in BC cell lines. *LHX3* overexpression significantly reduced the proliferation of BC as shown by crystal violet and colony formation assay. We also checked the impact of *LHX3* overexpression on the invasion, and migration of BC cells, which are critical early steps in cancer metastasis [48]. Overexpression of *LHX3* decreased the invasive and migratory capabilities of the BC cells. Reactome pathway analysis implied that *LHX3* is responsible for regulating SLITs and ROBOs, which are linked to processes like angiogenesis, cell migration, and metastasis in various cancers, including BC [49]. Furthermore, *LHX3* overexpression triggered DNA damage and apoptosis in these cancer cells. Notably, *LHX3* overexpression had a more pronounced inhibitory impact on MCF-7 compared to MDA-MB-231, which aligns with the known aggressive nature of MDA-MB-231 and its resistance to overexpression of various genes [50].

Finally, our efforts were directed towards uncovering the association between *LHX3* methylation and the clinicopathological characteristics of BC patients. Our data indicated significant associations between *LHX3* promoter methylation and metastasis status, tumor size, and TNM stage; however, these associations were not uniform across all indicators of disease progression. *LHX3* methylation was more frequent in smaller tumors and in tumors without distant metastasis, suggesting that this epigenetic alteration may occur in localized disease settings. However, methylation was also more frequent in advanced TNM-stage tumors. Because TNM stage is a composite clinical variable that incorporates tumor size, nodal involvement, and distant metastasis, this finding does not necessarily indicate a direct relationship between *LHX3* methylation and metastatic dissemination. Therefore, these clinicopathological data should be interpreted cautiously and should not be taken as evidence that *LHX3* methylation is a definitive early-stage marker or a progression/metastasis marker. Rather, the findings support *LHX3* as a candidate epigenetically regulated biomarker in BC. Future validation using independent cohorts and quantitative methylation approaches will be needed to establish the clinical utility of LHX3 methylation in BC.

In sum, our research marks the initial proof of the epigenetic suppression of *LHX3* in BC. Conversely, elevated LHX3 expression has been shown to diminish the viability and invasive potential of BC cells. Consequently, *LHX3* warrants further investigation as an epigenetically regulated tumor-suppressive candidate with potential biomarker relevance in BC. In the future, further experiments can be conducted to delve deeper into the mechanisms underlying *LHX3’*s regulation of breast tumorigenesis.

## Conclusion

In conclusion, this study identifies *LHX3* as an epigenetically silenced gene with tumor-suppressive properties in BC cells and supports its further investigation as a candidate epigenetic biomarker. However, its utility as a diagnostic or prognostic marker remains to be established through independent validation studies.

## Supporting information

S1 Fig *LHX3* promoter region; *LHX3 is* located at a minus strand of Chr 9 (q34.3) indicated by the red bar on the chromosomal map. It has two promotors; and we investigated the promotor corresponding to the transcript of interest (Ref Seq: NM_178138.5). A sequence around the 1000 bp upstream of transcription start site (TSS) and 200 bp downstream was obtained from UCSC on Human (GRCh38/hg38) Chr9:136,204,158−136,202,958 position.). Methprimer predicted two CpG islands on this sequence. The large island having (Transcription start site) TSS was investigated for methylation assays and the black arrow shows the primer location for MSP spanning the DNA from 136,203,346−136,203,238 bp.
(DOCX)

S1 Table List of downregulated and hypermethylated genes in breast tumor as compared to normal samples. Analysis was performed by using GEO2R tool. 40 hypermethylated and downregulated genes were obtained by overlapping these gene sets.
(XLSX)

S2 Table DAVID analysis to annotate the biological function to the list of 40 hypermethylated-downregulated genes. (XLSX)

S3 Table Shortlisted genes used for experimental analysis. Mean methylation and expression difference calculated by t-test value after GEO2R analysis.
(DOCX)

S1 Raw Images Full length images of gel electrophoresis used in this manuscript. (XLSX)

## Abbreviation:

BC: Breast cancer
CpG: 5’—C—phosphate—G—3’
DAVID: Database for Annotation, Visualization, and Integrated Discovery
DEG: Differentially Expressed Gene
DMG: Differentially Methylated Gene
EPD: Eukaryotic Promoter Database
GDC: Genomic Data Commons
GEO: Gene Expression Omnibus
IBC: Invasive Basal Carcinoma
IDC: Invasive Ductal Carcinoma
ILC: Invasive Lobular Carcinoma
LHX3: LIM homeobox 3
MSP: Methylation Specific PCR
OGs: Oncogenes
POC: point-of-care
qRT-PCR: Quantitative Real Time-Polymerase Chain Reaction
TSG: Tumor Suppressor Gene.
