# Supplementary material for: Integrative methylome and transcriptome analyses identify LHX3 as an epigenetically downregulated marker with tumor suppressive properties in breast cancer
Source: PLoS One. 2026 Oct 7;21(10):e0357378. doi: 10.1371/journal.pone.0357378 (PMC13645036; doi:10.1371/journal.pone.0357378)
Supplement: Supplementary file 1 [file pone.0357378.s001.docx]

**S1 Fig*:*** *LHX3* promoter region; *LHX3 is* located at a minus strand of Chr 9 (q34.3) indicated by the red bar on the chromosomal map. It has two promotors; and we investigated the promotor corresponding to the transcript of interest (Ref Seq: NM_178138.5). A sequence around the 1000bp upstream of transcription start site (TSS) and 200bp downstream was obtained from UCSC on Human (GRCh38/hg38) Chr9:136,204,158-136,202,958 position.). Methprimer predicted two CpG islands on this sequence. Large island having (Transcription start site) TSS was investigated for methylation assays and the black arrow shows the primer location for MSP spanning the DNA from136203457bp to136203012bp.
