# Supplementary material for: Integrative methylome and transcriptome analyses identify LHX3 as an epigenetically downregulated marker with tumor suppressive properties in breast cancer
Source: PLoS One. 2026 Oct 7;21(10):e0357378. doi: 10.1371/journal.pone.0357378 (PMC13645036; doi:10.1371/journal.pone.0357378)
Supplement: Supplementary file 2 [file pone.0357378.s004.docx]

**S3 Table**: Shortlisted genes used for experimental analysis. Mean methylation and expression difference calculated by t-test value after GEO2R analysis.

| **Gene symbol** | **NCBI**  **Accession no.** | **Title** | **Mean Methylation Difference** | **Mean Expression Difference** | ***P* value** | **Cellular Functions** |
| --- | --- | --- | --- | --- | --- | --- |
| *BHMT2* | [NM_017614.4](https://www.ncbi.nlm.nih.gov/entrez/query.fcgi?cmd=Search&db=Nucleotide&term=NM_017614&doptcmdl=GenBank&tool=genome.ucsc.edu) | betaine-homocysteine methyltransferase 2 | -5.7 | 3.4 | 1.78E-04 | - zinc ion binding - One-carbon metabolism and related pathways |
| *HLF* | [NM_002126](https://genome.ucsc.edu/cgi-bin/hgc?hgsid=656549711_vsUL1oKvA0fbV9sNEBvLY8ishPge&g=refGene&i=NM_002126&c=chr17&o=55265011&l=55265011&r=55325065&db=hg38) | HLF, PAR bZIP transcription factor | -6.28 | 5.45 | 1.91E-04 | - DNA binding - Positive regulation of transcription from RNA polymerase II promoter - Multicellular organism development |
| *LHX3* | NM 014564 | LIM homeobox 3 | -9.88 | 3.43 | 3.32E-15 | - DNA binding - Negative regulation of apoptotic process - Positive regulation of transcription - Motor neuron axon guidance |
| *KLK10* | [NM_002776.4](https://www.ncbi.nlm.nih.gov/entrez/query.fcgi?cmd=Search&db=Nucleotide&term=NM_002776&doptcmdl=GenBank&tool=genome.ucsc.edu) | kallikrein related peptidase 10 | -7.3 | 3.69 | 4.70E-06 | - MSP-RON Signaling |
| *CDC14B* | NM_033331 | cell division cycle 14B | -5.81 | 4.31 | 1.36E-04 | - Protein binding - Signal transduction |
| *AKAP12* | NM_005100 | A-kinase anchoring protein 12 | -7.17 | 6.38 | 6.46E-06 | - Protein binding - Signal transduction |
| *CCND2* | NM_001759 | cyclin D2 | -76.6 | 4.32 | 7.20E-09 | - Protein binding - Positive regulation of cell proliferation - Negative regulation of apoptotic process |
| *MAP3K7* | NM_145332 | mitogen-activated protein kinase kinase kinase 7 | -9 | 3.63 | 0.005962 | - ATP binding - transcription, DNA-templated |
